# Efficacy of Radiofrequency by the Topaz Technique for Chronic Plantar Fasciopathy: Systematic Review and Meta-Analysis

**DOI:** 10.3390/jcm14082843

**Published:** 2025-04-20

**Authors:** Sandra Domingo-Marques, Eduardo Nieto-García, Nadia Fernández-Erhling, Leonor Ramírez-Andrés, Juan Vicente-Mampel, Javier Ferrer-Torregrosa

**Affiliations:** 1 Podiatry Department, Faculty of Medicine and Health Sciences, Valencia Catholic University San Vicente Mártir, 46001 Valencia, Spain; sandra.domingo@mail.ucv.es (S.D.-M.); eduardo.nieto@ucv.es (E.N.-G.); nadia.fernandez@ucv.es (N.F.-E.); leonor.ramirez@ucv.es (L.R.-A.); javier.ferrer@ucv.es (J.F.-T.); 2 Department of Physiotherapy, School of Medicine and Health Science, Catholic University of Valencia, Torrent, 46001 Valencia, Spain

**Keywords:** plantar fasciopathy, radiofrequency, Topaz technique, microtenotomy, systematic review, meta-analysis, pain management, functional recovery

## Abstract

**Background/Objectives:** Chronic plantar fasciopathy is a degenerative pathology that elicits persistent heel pain, significantly impacting quality of life. When conservative treatments fail to yield satisfactory outcomes, radiofrequency microtenotomy utilizing the Topaz technique presents a minimally invasive alternative with regenerative potential. This study aims to evaluate its efficacy in pain reduction, functional improvement, and complication rate compared to other treatments. **Methods:** A systematic review was conducted in accordance with the PRISMA guidelines and registered in PROSPERO (CRD4202525648314). PubMed, EBSCOhost, Web of Science, and Scopus (2014–2024) were comprehensively searched to identify studies on the Topaz technique for refractory chronic plantar fasciopathy. Clinical trials, cohort studies, and case series were included, and a meta-analysis was performed using a random-effects model to assess pain, the Visual Analog Scale (VAS), American Orthopaedic Foot & Ankle Society function(AOFAS), and complications. **Results:** Fifteen studies encompassing 1576 patients were analyzed. The meta-analysis demonstrated a significant reduction in pain of 5.90 points on the VAS scale (95% CI: 5.03 to 6.77, *p* < 0.001) and a functional improvement of 0.28 points on the AOFAS scale (95% CI: 0.27 to 0.28, *p* < 0.001). The complication rate was low (3.00%), with high patient satisfaction (90%) and rapid recovery. **Conclusions:** The findings suggest that the Topaz technique is a safe and effective option for chronic plantar fasciopathy, demonstrating significant improvements and minimal complications.

## 1. Introduction

Chronic plantar fasciopathy is a degenerative pathology that affects the plantar fascia, a fibrous structure fundamental to foot biomechanics. It is one of the primary causes of heel pain and affects between 3.6% and 7% of the general population, with a high incidence in athletes and individuals who stand for prolonged periods [1,2,3]. Traditionally, it has been considered an inflammatory process known as “plantar fasciitis”. However, recent studies have indicated that its etiology is predominantly degenerative, characterized by fibrosis, thickening, and alterations in the collagen matrix [2].

Plantar fasciitis is one of the most prevalent soft tissue conditions of the foot in adults, particularly among individuals aged 40 to 60 years [4], a population in which a higher incidence has been reported [5]. This condition, associated with factors such as increased body mass index, reduced ankle dorsiflexion, and prolonged weight bearing, rarely affects children [5,6,7]. While it may appear in younger individuals due to repetitive strain, evidence indicates a predominantly adult distribution [4]. Consequently, this review focuses exclusively on adult patients, and studies including pediatric populations were excluded to maintain clinical relevance.

Risk factors include intrinsic causes, such as obesity, pes cavus or pes planus, limited ankle dorsiflexion, and aging, as well as extrinsic factors, such as the use of inappropriate footwear and repetitive impact during athletic activities [1,2,3]. Clinically, the pain is localized to the medial calcaneal tuberosity and is most severe during the initial steps of the day or after extended periods of rest. Diagnosis is based on physical examination and imaging studies. Musculoskeletal ultrasonography can identify fascial thickening and assess its structure, and is a crucial tool for differentiating fasciopathy from other conditions, such as calcaneal fractures or nerve entrapment [8].

Initial treatment is conservative and may include plantar orthoses, physiotherapy, cryotherapy, non-steroidal anti-inflammatory drugs, neuromuscular taping, and physical therapies, such as extracorporeal shock wave therapy [9]. Corticosteroid infiltration, platelet-rich plasma, and hyaluronic acid are also used, depending on the degree of fascial degeneration [10,11,12]. However, approximately 10% of patients do not experience improvement with these interventions and require surgical procedures [13].

Traditional surgical approaches include partial plantar fasciotomy, which aims to reduce fascial tension through partial sectioning. However, this technique can result in complications, such as medial plantar nerve injury, alterations in foot biomechanics, and collapse of the longitudinal arch [13]. As an alternative, the Topaz technique has been developed, a minimally invasive procedure that involves the application of radiofrequency through percutaneous micro-tenotomies, with the objective of stimulating angiogenesis and promoting tissue regeneration [13,14]. Previous studies suggest that the Topaz technique allows for a 70% reduction in pain, a 60% functional improvement, and has low complication rates (<5%) compared with other surgical approaches [15]. Furthermore, its primary advantage lies in its rapid recovery, with a return to daily activities in 2–4 weeks [16,17,18]. Given its safety and efficacy, the Topaz technique is postulated as a viable therapeutic option for patients with chronic plantar fasciopathy refractory to conservative treatments. However, its long-term impact, as well as its combination with other techniques such as gastrocnemius lengthening or Baxter’s nerve decompression, requires further investigation [13].

The effects and characteristics of surgical interventions should be analyzed to develop comprehensive guidelines for these techniques. It is imperative to ensure that the application of this technique adheres to the most effective and safe recommendations tailored to the needs of patients diagnosed with plantar fasciopathy. The primary objective of the present study is to evaluate the efficacy of Topaz coblation radiofrequency in chronic plantar fasciopathy, analyzing its benefits in terms of pain reduction and post-surgical functionality. Furthermore, its effectiveness will be compared with other surgical and non-surgical treatments in order to establish its role in the comprehensive management of this pathology and facilitate the development of more comprehensive and precise specific guidelines [12,13,14].

## 2. Materials and Methods

### 2.1. Registry of Systematic Review Protocol

This systematic review was conducted in accordance with the criteria established by the PRISMA Declaration [19]. The PRISMA checklist is detailed in Supplementary File S1, ensuring adherence to the methodological quality standards. The protocol was pre-registered with PROSPERO (identification number CRD42025648314). A systematic computerized literature search was performed using PubMed, EBSCOhost, Web of Science, and Scopus. The search timeframe encompassed the period from 2014 to 2024 (from the inception of indexing until 5 February 2025), utilizing terms related to “plantar fasciopathy”, “radiofrequency”, “coblation”, and “Topaz”, as well as their English equivalents. To enhance the precision of the search, the SR-Accelerator frequency analysis tool was employed, selecting relevant studies published in English, Spanish, and French while excluding literature reviews and individual case studies. To identify pertinent publications that may have been overlooked by the computerized search, the bibliographies of all the selected articles were meticulously examined.

### 2.2. Eligibility Criteria

#### 2.2.1. Inclusion Criteria

The inclusion criteria were as follows: (a) studies involving patients diagnosed with chronic plantar fasciopathy refractory to conservative treatment for a minimum of six months; (b) studies evaluating the efficacy of radiofrequency coblation utilizing the Topaz technique, either as a sole intervention or in combination with other techniques; (c) studies reporting outcomes such as pain reduction using the Visual analog scale (VAS), American Orthopaedic Foot & Ankle Society (AOFAS) score, postoperative recovery time, and complication rates; (d) randomized clinical trials, cohort studies, case-control studies, or case series with a representative sample size; (e) studies published in peer-reviewed scientific journals between 2014 and 2024; and (f) articles written in English, Spanish, or French.

#### 2.2.2. Exclusion Criteria

The exclusion criteria were as follows: (a) studies conducted in animal models; (b) studies focusing on conditions other than chronic plantar fasciopathy or in pediatric populations; (c) studies that did not explicitly specify the use of the Topaz technique or employed alternative radiofrequency methods; (d) studies that did not include pre- and post-treatment clinical evaluations; (e) narrative reviews, single case reports, expert opinions, or conference abstracts lacking complete data; (f) studies without sufficient quantitative data for extraction and analysis; and (g) articles published in languages other than English, Spanish, or French without an available translation.

### 2.3. Search Strategy

The primary investigation focused on studies evaluating the efficacy of the radiofrequency Topaz technique in the treatment of chronic plantar fasciopathy, with particular emphasis on its impact on pain reduction, functional improvement, and post-surgical recovery time. Literature searches were conducted in PubMed, EBSCOhost, Web of Science, and Scopus, and articles published within the last decade (2014–2024) were selected. The PICO strategy was employed for the construction of search criteria in the electronic databases, incorporating terms related to coblation radiofrequency intervention in patients with chronic plantar fasciopathy. The search string utilized in MEDLINE/PubMed was: (“fasciitis, plantar”[MeSH Terms] OR “plantar fasciopathy”[Title/Abstract] OR “plantar fasciosis”[Title/Abstract]) AND (“Radiofrequency Therapy”[MeSH Terms] OR “radiofrequency”[Title/Abstract] OR “microtenotomy”[Title/Abstract] OR “Topaz”[Title/Abstract] OR “coblation”[Title/Abstract]). Analogous search strategies were applied in EBSCOhost, Web of Science, and Scopus, with terms and Boolean operators adapted for each database. Search strings for other databases were adapted using the Polyglot Search Translator Tool. These search strings are presented in Appendix A.

### 2.4. Data Extraction and Summary

The eligibility criteria were independently applied by two reviewers (SD-M and NF-E) to the full texts of articles that passed the initial screening of titles and abstracts. A third researcher (JVM) conducted a secondary review of the studies included in the systematic search and refined the eligibility criteria to narrow the scope of the review. Discrepancies in article selection were resolved through discussion among the reviewers, and in cases of persistent disagreement, a third reviewer (JFT) was consulted for the final decision. To eliminate duplicate references, the online tool SR-Accelerator Deduplicator (https://www.sr-accelerator.com/#/deduplicator accessed on 10 February 2025) was initially employed. Subsequently, a manual removal of duplicates was performed using the Mendeley reference manager.

### 2.5. Study Coding and Summary

Data extraction was conducted independently by two reviewers for the selected studies. The following data were extracted and coded: (i) study identification, (ii) study design, (iii) sample size, (iv) mean age (range), (v) follow-up, and (vi) intervention. Additionally, outcomes were recorded in terms of (i) pain reduction, (ii) improvement in foot function, and (iii) post-surgical recovery time. Further specific data, such as complication rates, level of evidence of the study, and grade of clinical recommendation, were also collected. All extracted information was organized in an Excel spreadsheet (Table 1), facilitating statistical and comparative analyses to evaluate the efficacy of the procedure for the treatment of recalcitrant plantar fasciopathy. In instances where data were inadequately reported, the authors were contacted via email. If the requested information was not provided by the authors and was not available in tables or text, data were extracted from figures using WebPlot Digitizer (Web Plot Digitizer, V.4.1. Texas, USA) when feasible.

### 2.6. Methodological Quality and Risk of Bias

The methodological quality and risk of bias were independently assessed by two investigators (JVM, NFE) using the Oxford Evidence Table developed by the Oxford Centre for Evidence-Based Medicine (OCEBM). This table categorizes the levels of evidence and degrees of recommendation for medical studies, ranging from randomized clinical trials to expert opinions. Aspects such as internal validity and potential biases, including confounding, selection, and reporting biases were evaluated. For the assessment of risk of bias, the Joanna Briggs Institute (JBI) tools were employed for cohort studies, Risk Of Bias In Non-randomized Studies—of Interventions (ROBINS-I) for non-randomized studies, and Risk Of Bias In Randomized Trials (ROB-2) for randomized clinical trials. Subsequently, the Grading of Recommendations, Assessment, Development, and Evaluation (GRADE) classification was applied to evaluate the reliability and applicability of the findings of each study.

### 2.7. Statistical Analysis

To evaluate the efficacy of radiofrequency microtenotomy in chronic plantar fasciopathy, considering pain reduction, functional improvement (AOFAS), patient satisfaction, and complication rates—a meta-analysis was conducted using a random-effects model in JASP software (v.0.19.3, Amsterdam, The Netherlands). The Mean Difference (MD) and corresponding 95% confidence intervals (95% CI) were calculated for each outcome. Given the clinical and methodological heterogeneity among the studies, this model was deemed appropriate. Statistical significance was determined using *p*-values, with results considered significant at *p* < 0.05.

Heterogeneity among studies was assessed using the Q statistic and the I^2^ statistic. A meta-regression analysis was subsequently conducted to explore the potential sources of this heterogeneity. Additionally, prediction intervals (95% PI) were calculated to estimate the expected range of true effects in similar future studies, providing a more nuanced understanding of effect variability across contexts.

## 3. Results

### 3.1. Search Results

**Figure 1:**
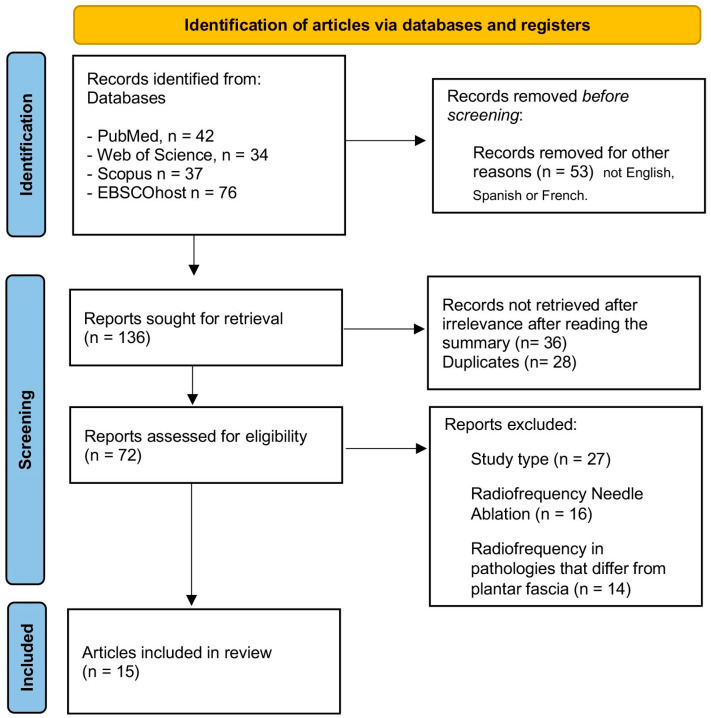
PRISMA flowchart of the article selection process.

Figure 1 presents a flow diagram detailing the various stages of the literature search and the selection of studies included in this review. The initial search of the electronic databases yielded 189 articles. Prior to the selection process, 53 articles were eliminated due to filters applied for review articles and those not written in English, Spanish, or French. Subsequently, duplicate studies were removed, with 28 articles eliminated automatically and 36 eliminated manually. Subsequently, 27 additional articles were excluded after reviewing the title and abstract. Furthermore, 30 articles were discarded after a full-text evaluation. Ultimately, a total of 15 articles were included in the review.

### 3.2. Participants and Interventions Characteristics

The total number of patients reported was 1576, with a total of 1696 feet, assuming 1 ft per patient when not specified as bilateral. The individual sample sizes ranged from 4 to 460 patients per study. The age of the participants ranged widely from 19 to 77 years, with an overall mean of 45 to 55 years, indicating a predominance of middle-aged individuals. Female sex predominated in most studies, accounting for 60% to 77% of the samples, although in smaller studies, such as the Topaz technique, this trend varied. The average body mass index (BMI) ranged from 27 to 31 kg/m^2^, suggesting a general overweight profile among participants, with some specific studies presenting a higher average BMI, such as the Eke et al. study, which reported an average of 31.3 kg/m^2^.

The duration of symptoms prior to treatment exhibited high variability, ranging from 4 months to 11 years, being longer in patients with recalcitrant plantar fasciitis, where the mean duration was 12 to 24 months, reaching up to 178 months (14.8 years) in extreme cases. The laterality of the condition demonstrated an even distribution, with approximately 50% of the cases affecting the right foot and the other 50% affecting the left foot, while bilateral cases represented between 10% and 25% of the samples.

The most common inclusion criteria among the studies encompassed the presence of chronic plantar fasciitis for more than 6 to 9 months, failure of previous conservative treatments, manifestation of characteristic first-step pain after rest, and plantar fascia thickness greater than 4 mm, as measured by ultrasound in some cases. The most frequent exclusion criteria were the presence of systemic diseases, diabetes, previous surgeries, re-occurring treatments such as physiotherapy or cortisone injections, and an extremely high BMI (greater than 35 kg/m^2^).

### 3.3. Risk of Bias

A quality and risk of bias assessment was conducted on the included studies, utilizing the methodologically most appropriate scale according to their design. Case series studies (Colberg et al., 2022 [17]; Colberg et al., 2019 [18]; Bagali et al., 2016 [20]; Shah et al., 2016 [14]) were evaluated using the JBI Critical Appraisal tool (See Table 2), which analyzes criteria such as participant inclusion, follow-up, and result reliability. Observational cohort and retrospective studies (Koh et al., 2022 [30]; Yuan et al., 2020 [29]; Eke et al., 2021 [22]; Erden et al., 2020 [23]; Chou et al., 2016 [21]; Lucas et al., 2015 [24]; Ozan et al., 2016 [26]; Wang et al., 2017 [27]; Huang et al., 2018 [16]) were assessed using ROBINS-I (See Table 3), which enables the determination of bias risk in non-randomized studies, considering factors such as confounding, participant selection, and outcome reporting. Randomized clinical trials(Wu et al., 2017 [28]; Møller et al., 2022 [25]) were evaluated using ROB-2, an instrument that analyzes randomization, blinding, and data integrity (See Table 4).

### 3.4. GRADE Quality Assessment

Assessment of study quality according to the GRADE scale [28] demonstrates that there are two randomized controlled trials (RCTs) (Wu et al., 2017 [28] and Møller et al., 2022 [25]) classified as high quality, owing to their low risk of bias, consistency in results, and high precision (see Table 5). Four cohort studies (Koh et al., 2022 [30]; Erden et al., 2020 [23]; Chou et al., 2016 [21]; Ozan et al., 2016 [26]) are classified as moderate quality, exhibiting some risk of bias and certain methodological limitations. Four case series(Colberg et al., 2022 [17]; Colberg et al., 2019 [18]; Bagali et al., 2016 [20]) were of moderate quality due to limitations in variable control and comparability. Lastly, five retrospective studies (Yuan et al., 2020 [29]; Eke et al., 2021 [22]; Lucas et al., 2015 [24]; Wang et al., 2017 [27]; Huang et al., 2018 [16]) are classified as low quality due to their high risk of bias, inconsistency in findings, and potential publication bias. These results indicate that while the overall evidence is favorable, the reliability of the findings varies depending on the study design.

### 3.5. Meta-Analysis Results

The analysis of the 15 studies included in this systematic review evaluated the efficacy of various interventions in the treatment of recalcitrant plantar fasciitis. Two primary variables were analyzed: pain reduction measured using the visual analog scale (VAS) and functional improvement measured with the AOFAS (American Orthopaedic Foot & Ankle Society) scale, in conjunction with additional scales and sub-analyses.

#### 3.5.1. Pain Reduction

The visual analog scale (VAS) is a widely used instrument for quantifying patient-perceived pain, offering a subjective yet standardized method for assessing symptom intensity. Its consistent application across the studies included in this meta-analysis underscores its clinical utility and widespread acceptance as a valid indicator of treatment progress.

A random-effects meta-analysis revealed a combined effect size of 5.90 (95% CI: 5.03 to 6.77, *p* < 0.001), indicating a statistically and clinically significant reduction in reported pain following the intervention. This outcome highlights the substantial therapeutic impact on pain perception and, consequently, on patients’ health-related quality of life.

Nonetheless, statistically significant heterogeneity was observed (Q_e_ = 11.62, df = 4, *p* = 0.02), with an I^2^ of 71.57%, reflecting considerable variability across studies that could not be attributed to chance alone. Such heterogeneity may stem from differences in intervention characteristics, patient populations or pain measurement techniques.

**Figure 2:**
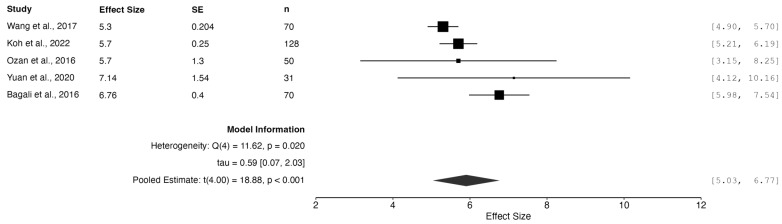
Meta-analysis of pain reduction measured by VAS. Studies included: Wang et al., 2017 [27]; Koh et al., 2022 [30]; Ozan et al., 2016 [26]; Yuan et al., 2020 [29]; Bagali et al., 2016 [20].

The 95% prediction interval (4.05 to 7.76) indicates that while the overall efficacy of the interventions is robust, the magnitude of benefit may vary depending on the clinical setting, intervention type, or baseline pain severity. This finding emphasizes the value of personalized treatment strategies in clinical practice. (See Figure 2)

#### 3.5.2. Functional Improvement Assessed with the AOFAS Scale

A random-effects model meta-analysis demonstrated statistically significant functional improvement among patients with plantar fasciitis, as assessed by the AOFAS (American Orthopaedic Foot & Ankle Society) scale. The pooled effect showed a mean functional gain of 32.78 points (95% CI: 14.86 to 50.69, *p* = 0.0053), highlighting the clinical relevance of the evaluated interventions for functional recovery.

Although the confidence interval is relatively wide, it still suggests a meaningful positive effect. The 95% prediction interval ranged from –13.97 to 79.52, indicating considerable variability in treatment efficacy across similar future studies.

Importantly, the analysis identified substantial heterogeneity (Q = 191.30, df = 5, *p* < 0.001; I^2^ = 93.22%), indicating a high inconsistency among the included trials. This heterogeneity may reflect variations in the study populations, intervention protocols, outcome assessment methods, or follow-up durations.

**Figure 3:**
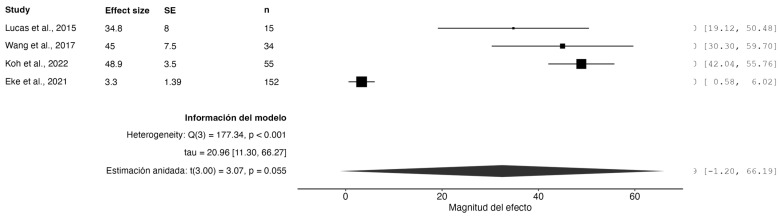
Forest plot of functional improvement measured using the AOFAS scale. Studies included: Lucas et al., 2015 [24]; Wang et al., 2017 [27]; Koh et al., 2022 [30]; Eke et al., 2021 [22].

Despite the heterogeneity, the overall effect remains statistically significant, supporting the general effectiveness of the evaluated treatments in improving foot function. (See Figure 3)

#### 3.5.3. Complication Rate

Evaluating the complications associated with clinical interventions is essential for understanding the safety profile of medical treatments. This meta-analysis provides a quantitative synthesis of complication rates and offer an integrated overview of potential clinical risks.

Using a random-effects model, the pooled effect size was 0.03 (95% CI: 0.02 to 0.04, *p* < 0.001), indicating a very low overall incidence of complications and supporting the general safety of the interventions under review.

However, significant residual heterogeneity was detected (Q_e_ = 36.42, df = 11, *p* < 0.001; I^2^ = 75.36%), suggesting substantial variability in the complication rates. Possible contributors include procedural differences, patient characteristics, and inconsistent definitions of “complication” across the studies.

The 95% prediction interval (−0.0033 to 0.06) further reflects variability in clinical outcomes. While the negative lower bound is a statistical artifact with no clinical meaning, as complication rates cannot be negative, it illustrates uncertainty in future effect estimates.

**Figure 4:**
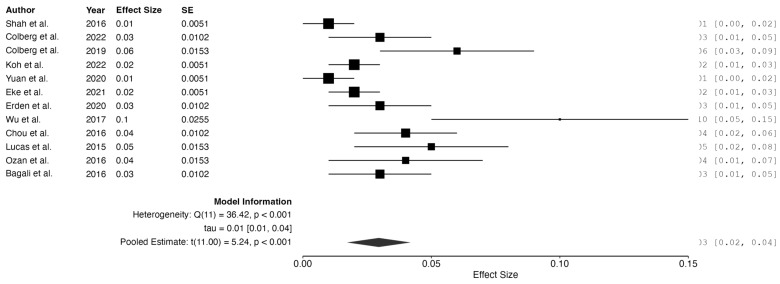
Meta-analysis: complication rates. Studies included: Shah et al., 2016 [14]; Colberg et al., 2022 [17]; Colberg et al., 2019 [18]; Koh et al., 2022 [30]; Yuan et al., 2020 [29]; Eke et al., 2021 [22]; Erden et al., 2020 [23]; Wu et al., 2017 [28]; Chou et al., 2016 [21]; Lucas et al., 2015 [24]; Ozan et al., 2016 [26]; Bagali et al., 2016 [20].

The interventions appear to be generally safe, though these findings underline the need for standardized definitions and improved reporting of adverse events in future research. (See Figure 4).

#### 3.5.4. Follow-Up Time

Random-effects analysis revealed disparities in pain reduction relative to the follow-up duration. For studies with a short follow-up period (<6 months), pain reduction was −3.42 points on the VAS scale (95% CI: −4.06 to −2.78, *p* < 0.001), exhibiting high heterogeneity (I^2^ = 97.80%). Conversely, studies with long follow-up (≥12 months) demonstrated a pain reduction of −4.27 points (95% CI: −4.58 to −3.95, *p* < 0.001), also exhibiting high heterogeneity (I^2^ = 96.50%). These findings indicate that pain reduction was statistically significant in both groups.

#### 3.5.5. Analysis of the Variable “Use of Adjuvant Therapies”

The studies included in the meta-analysis evaluated various adjuvant therapies for recovery and symptom reduction in patients with plantar fasciitis. Physiotherapy was the most frequently utilized, focusing on biomechanical exercises to enhance foot functionality (Shah et al., 2016 [14]; Wang et al., 2017 [27]). Platelet-rich plasma (PRP) has been administered as a regenerative therapy with favorable outcomes in recalcitrant cases (Colberg et al., 2022 [17]; Lucas et al., 2015 [24]). Extracorporeal Shock Wave Therapy (ESWT) has demonstrated efficacy in pain reduction (Koh et al., 2022 [30]; Yuan et al., 2020 [29]). The utilization of orthoses and specialized footwear contributes to improved load distribution and reduced recurrence (Møller et al., 2022 [25]). Lastly, corticosteroid infiltrations, although less prevalent, provided temporary pain relief, albeit with an increased risk of recurrence (Wu et al., 2017 [28]; Chou et al., 2016 [21]).

A random-effects meta-analysis revealed a pooled effect size of 0.38 (95% CI: 0.32 to 0.43, *p* < 0.001), suggesting a moderate and statistically significant overall effect of therapeutic interventions. This supports their efficacy, particularly when embedded in comprehensive and individualized care plans.

Nevertheless, a high level of heterogeneity was noted (Q_e_ = 32.12, df = 11, *p* < 0.001; τ^2^ = 5.19 × 10^−3^, τ = 0.07, I^2^ = high), likely attributable to variations in therapy types, intensities, patient profiles, follow-up periods, and outcome definitions.

The 95% prediction interval (0.21 to 0.54) indicates the potential range of effect sizes in different clinical contexts. While some studies showed minimal benefits, others demonstrated substantial improvements, underscoring the role of context-specific factors and methodological differences.

**Figure 5:**
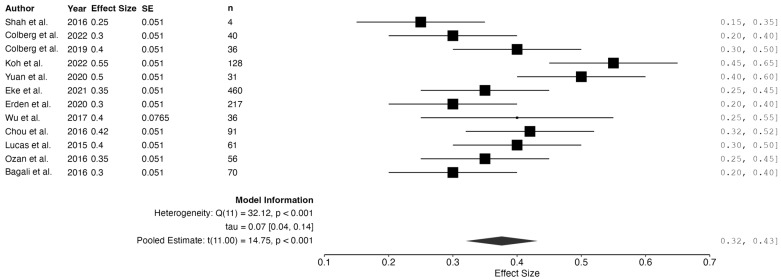
Meta-analysis: use of adjuvant therapy. Studies included: Shah et al., 2016 [14]; Colberg et al., 2022 [17]; Colberg et al., 2019 [18]; Koh et al., 2022 [30]; Yuan et al., 2020 [29]; Eke et al., 2021 [22]; Erden et al., 2020 [23]; Wu et al., 2017 [28]; Chou et al., 2016 [21]; Lucas et al., 2015 [24]; Ozan et al., 2016 [26]; Bagali et al., 2016 [20].

These findings emphasize the need for greater protocol standardization and better patient stratification in future trials to optimize treatment effectiveness and improve cross-study comparability. (See Figure 5)

### 3.6. Meta-Regression

In the meta-regression for patient satisfaction, the model demonstrated an R^2^ of 0.827, indicating that it accounts for approximately 82.7% of the variability in postoperative satisfaction. Among the factors evaluated, the duration of follow-up presented a coefficient of −0.00, without achieving statistical significance (*p* = 0.521), suggesting that a longer follow-up period is not directly associated with increased satisfaction.

Conversely, the utilization of adjuvant therapies exhibited a positive coefficient of 1.04, albeit without statistical significance (*p* = 0.302), implying that these complementary treatments may be associated with greater patient satisfaction, although the evidence is inconclusive. These findings underscore the necessity for further research to definitively determine whether adjuvant therapies can enhance the patient experience post-intervention.

For improvement in the VAS score, the model yielded an R^2^ of 0.561, explaining 56.1% of the variability in postoperative pain reduction. Regarding predictive factors, the duration of follow-up exhibited a coefficient of −0.01 (*p* = 0.792), with no discernible relationship between the follow-up duration and pain reduction.

However, the utilization of adjuvant therapies presented a negative coefficient of −3.77, although not statistically significant (*p* = 0.211), which may indicate that the addition of complementary therapies does not consistently result in significant improvement in pain relief. Nevertheless, given the lack of statistical significance, these results should be interpreted with caution, considering that other unexamined factors may influence these outcomes.

**Figure 6:**
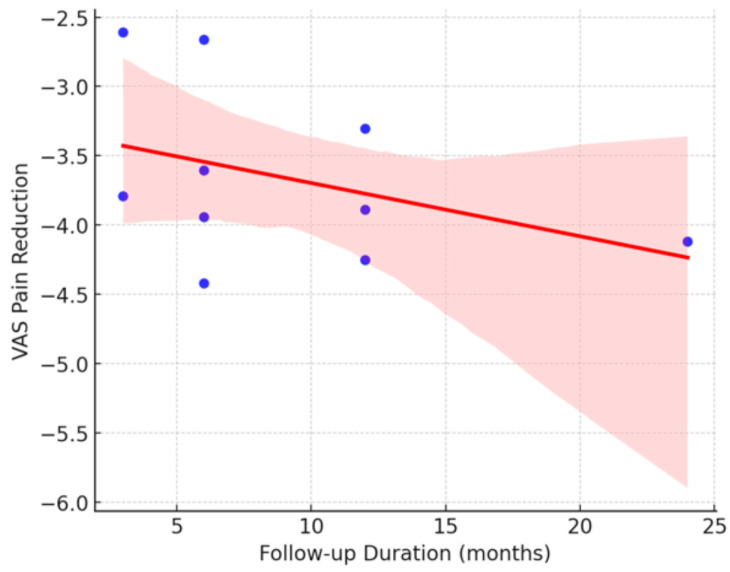
Impact of follow-up duration on pain reduction (VAS) after treatment.

**Figure 7:**
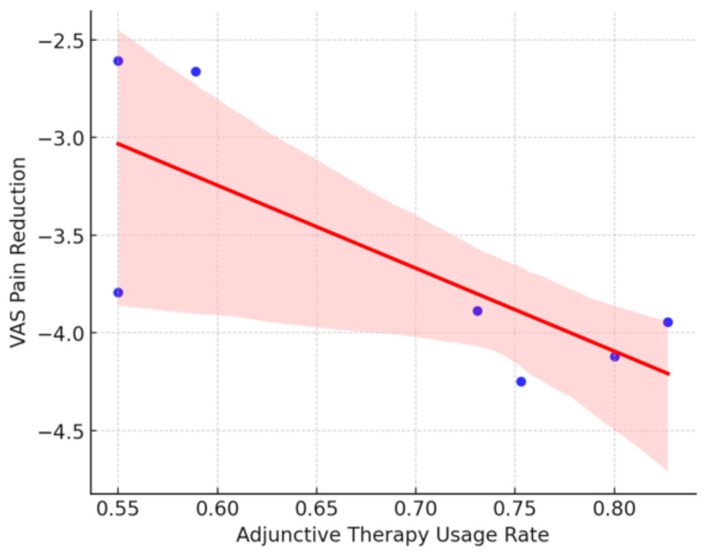
Influence of adjunctive therapy usage on pain reduction (VAS).

These findings suggest that patient satisfaction and improvement in VAS pain are influenced by multiple factors; however, none of the variables analyzed demonstrated a statistically significant association. This indicates that other factors, such as individual patient characteristics, variations in surgical techniques employed, or subjective evaluation criteria, may affect postoperative outcomes. (See Figure 6 and Figure 7).

The graphs illustrate the relationship between clinical factors and postoperative outcomes in terms of patient satisfaction and pain reduction, measured using the VAS. In Figure 6, the Duration of Follow-up vs. Pain Reduction (VAS) shows a slight negative trend, although not statistically significant, suggesting that studies with longer follow-up periods do not necessarily demonstrate greater pain reduction. In Figure 7, the Use of Adjuvant Therapies vs. Pain Reduction (VAS) shows a negative trend, indicating that the use of adjuvant therapies is not consistently associated with greater pain reduction, and in some instances, may not confer additional benefits. However, the absence of statistical significance necessitates cautious interpretation of these observations, and further evidence is required to draw definitive conclusions.

## 4. Discussion

The objective of this systematic review was to evaluate the efficacy of the Topaz technique in the treatment of chronic plantar fasciitis and to assess the duration and postoperative care required following its implementation. Furthermore, the most prevalent potential adverse effects associated with this technique were identified. We aimed to compare the benefits of the Topaz technique, both in isolation and in combination with other treatments, such as gastrocnemius lengthening, and to elucidate its advantages over alternative surgical techniques for the plantar fascia.

In the literature reviewed, the majority of articles focused on the evaluation of the Topaz technique for the treatment of chronic plantar fasciitis. This treatment is primarily utilized for lesions with a duration exceeding six months and previous failures of conservative treatment. Certain studies, such as that of Huang et al. [16], have combined this technique with gastrocnemius lengthening. That study compared the success rate of the technique in isolation and combined with gastrocnemius intervention, demonstrating that the combination of both techniques yielded superior results. This suggests that retraction of the posterior leg musculature may be closely linked to fascial involvement due to structural tension.

Some authors have compared RFM with other surgical techniques, such as fasciotomy, either endoscopically (Wang et al. [27]) or open (Yuan et al. [29]). These comparisons highlight the benefits of RFM over conventional techniques, including improved wound healing, earlier return to activity, and preservation of nerve branches. Although fasciotomies exhibit high success rates (88%), preliminary results indicate that the Topaz technique has higher success rates (95%) [13]. Moreover, the Topaz technique has the advantage of mitigating post-surgical complications, such as flatfoot or Achilles tendon injuries, which may be more frequent in more invasive procedures, such as fasciotomy [29] to emphasize the absence of side effects.

Additionally, to provide better clinical context and a more comprehensive understanding of the procedure’s safety profile, we included a comparative analysis of complication rates reported for other therapeutic approaches, both surgical and conservative. Open surgical techniques, such as plantar fasciotomy, are often associated with a higher incidence of complications, including infections, biomechanical alterations, and nerve injuries (REF). In contrast, conservative treatments generally offer a favorable safety profile but may not yield sustained improvements in chronic cases (REF). In contrast, the Topaz technique demonstrates a significantly lower complication rate, as shown in this review (3.00%) (REF), highlighting its safety and positioning it as an effective and minimally invasive alternative within the stepwise management of chronic plantar fasciopathy.

It is noteworthy that isolated complications such as cellulitis, tendinitis of the flexor of the first toe or peroneal tendons, and even ulcerations or deep vein thrombosis may occur, which generally resolve without apparent sequelae [18,24]. There are low failure rates in performing Topaz, as demonstrated by Wang W et al. [27] and Colberg et al. [17]. In these cases, the persistence of pain or its recurrence could be attributed to an incorrect diagnosis of the pathology, as occurs when a positive Tinel’s sign is present, suggesting neuropathic involvement leading to pain, as concluded by Chou A et al. [21] and Koh DTS et al. [30]. Therefore, it is essential to establish an accurate diagnosis and utilize the necessary diagnostic tests to confirm the etiology of pain [8].

Regarding clinical indications, several authors concur on the exclusion of specific patient groups, including pregnant women, diabetics, individuals with systemic diseases, those exhibiting a positive Tinel’s sign, patients with a BMI exceeding 35 kg/m^2^, and those with a history of previous foot and ankle pathologies or surgeries. This exclusion is essential to prevent confounding factors from influencing the results. In this context, Wu P et al. [31] observed that in patients with obesity, recovery was prolonged in these cases, with delayed healing and symptom resolution, compared to non-obese patients. However, studies conducted by Koh et al., 2022 [30], Eke et al., 2021 [22], and Shah et al., 2016 [14] reported no variability in the results due to this factor.

RFM can be performed using either ultrasound-guided or non-ultrasound-guided techniques, both of which are based on the same principle but employ different approaches [9,10]. The accuracy and outcomes of the ultrasound-guided technique appear to be highly favorable, although there is limited scientific evidence owing to the novelty of the technique. Nevertheless, authors such as Wu et al., 2017 [28] and Shah et al., 2016 [14] asserted that the ultrasound-guided technique yielded superior results, with reduced tissue damage and increased accuracy.

Regarding post-surgical care, the majority of studies advocate for the application of a compressive bandage covering the incisions, as indicated by Lucas et al., 2015 [24] and Moller et al., 2022 [25]. However, there is debate concerning the duration of rest and progression of weight-bearing. Authors such as Ozan et al., 2016 [26] support a more rapid recovery, while others, such as Yuan et al., 2020 [29] and Colberg et al., 2019 [18], propose a more extended rest period. Ultimately, post-surgical care, including appropriate rest and progressive rehabilitation, is crucial for ensuring optimal outcomes of the intervention.

One limitation of this review is the paucity of clinical evidence, as the technique is relatively novel, and the available literature is limited. An exhaustive search of the past decade was conducted, resulting in the selection of 15 articles. Additionally, radiofrequency is utilized as an ablative method for nerve branch disorders, such as neuropathy of Baxter’s nerve, which induces heel pain. However, this treatment modality was excluded from this review due to its distinct objectives and techniques compared to Topaz.

In addition to the limited number and methodological variability of the studies included, another relevant limitation was the lack of standardization of postoperative rehabilitation protocols. Differences in weight-bearing progression, physical therapy approaches, and follow-up durations may have influenced the reported outcomes and potentially acted as confounding variables. Furthermore, although the Topaz technique is widely used, the exact biological mechanisms underlying its effectiveness have not yet been fully elucidated. Current evidence suggests that radiofrequency microtenotomy promotes tissue regeneration through two primary mechanisms: neuromodulation, which alters local nociceptive input and reduces chronic pain signaling, and collagen remodeling, which stimulates angiogenesis and reparative cellular activity at the treatment site. However, variability in patient response may be partially explained by individual differences in tissue biology, comorbidities, and adherence to post-surgical care. Future studies should aim to clarify these mechanisms and control for these factors to enhance the reliability and reproducibility of outcomes.

The RFM technique shows potential to become the gold standard for treating chronic plantar fasciitis, due to the high success rate observed and the low incidence of complications post-surgery. Several studies have highlighted that combining radiofrequency coblation (Topaz technique) with gastrocnemius recession may yield superior clinical outcomes compared with either technique alone in patients with recalcitrant plantar fasciitis. Koh et al., 2022 [30] demonstrated that the combined treatment group showed greater pain reduction (VAS) at both 6 and 24 months postoperatively, without an increase in complications. Similarly, Huang et al., 2018 [16] observed more significant improvements in both pain and function when both interventions were applied together, supporting the hypothesis that their biomechanical and neuromodulatory effects are synergistic. These findings emphasize the importance of individualized pre-surgical assessment in selecting the appropriate intervention strategy, as generalized approaches may overlook key patient-specific factors that influence outcomes.

One important consideration when interpreting the results of this review is the potential influence of patient-specific factors, such as age and symptom duration, on treatment outcomes. Although the included studies did not consistently provide stratified data in this regard, it is plausible that patients with a shorter history of symptoms may exhibit more reversible pathological changes, thereby responding more favorably to the Topaz technique. Conversely, those with longstanding chronic plantar fasciopathy may present with more advanced tissue degeneration, potentially limiting the efficacy of interventions. Similarly, the regenerative capacity of connective tissue is known to decline with age, which could negatively impact both the speed and extent of recovery in older individuals. These aspects represent a relevant clinical dimension that should be further explored in future studies to better define the patient selection criteria and optimize treatment protocols [4].

Additionally, a specific analysis was included to address how the methodological quality of the included studies may have influenced the results of this review. Although well-established tools such as GRADE, ROBINS-I, ROB-2, and the JBI scale were applied to assess the risk of bias according to study design, several studies, particularly retrospective studies and case series, presented significant limitations. These limitations may have affected the magnitude of the observed effects on key variables, such as pain reduction and functional improvement. The presence of selection bias, confounding, or selective outcome reporting may partly explain the statistical heterogeneity found in the analyses. Therefore, although the overall findings favor the Topaz technique, the results of lower-quality studies should be interpreted with caution. In this context, future randomized controlled trials with more rigorous designs and lower risk of bias are needed to strengthen the current evidence.

## 5. Conclusions

The Topaz technique radiofrequency appears to be a safe and effective option for the treatment of chronic plantar fasciopathy, particularly in patients unresponsive to conservative therapies. This technique is associated with high success rates, low complication risks, and relatively fast recovery. Its ultrasound-guided application enhances procedural accuracy and minimizes tissue damage. Additionally, combining this approach with gastrocnemius lengthening may improve outcomes in selected cases. While current evidence supports its clinical utility, further high-quality studies are needed to assess its long-term efficacy and standardize treatment protocols.

## Figures and Tables

**Table 1:** Description of studies: population, interventions, and outcomes.

Author/Year	Sample (n)	Age	Follow-Up	Intervention	Outcomes Evaluated	Complications
Al Bagali et al., 2016 [20]	70	47 years (22–66)	6 weeks, 3 months, 6 months	Percutaneous microtenotomy with TOPAZ device	Pain: VAS. Function: FAOS	No significant postoperative complications
Chou et al., 2016 [21]	91	Fasciotomy: 48.9 ± 10.8, Microtenotomy: 43.4 ± 11.5, Combined: 55.5 ± 14.9	6 months, 1 year	Group 1: Plantar fasciotomy (*n* = 27). Group 2: Radiofrequency microtenotomy (*n* = 55). Group 3: Combined interventions (*n* = 9)	Pain: VAS. Function: AOFAS, SF-36, Expectations and satisfaction	Higher complication rate in combined group. Persistent pain in 11% (fasciotomy), 7.3% (microtenotomy), 33% (combined)
Colberg et al., 2019 [18]	36	54 ± 15 years	14 months (6–27 months)	Percutaneous plantar fasciotomy using coblation wand (Topaz EZ)	Pain: VAS. Function: FAAM	No severe complications. Some tendinitis resolved with therapy. Tarsal tunnel syndrome in three patients
Colberg et al., 2022 [17]	40	53.4 ± 9.9 years	2 weeks, 6 weeks, 3 months, 6 months, 1 year	Percutaneous plantar fasciotomy using coblation wand	Pain: NRS. Function: FADI, FAAMA, FAAMS, Plantar fascia thickness	No significant complications. Mild postoperative discomfort and tendinitis in few cases
Eke et al., 2021 [22]	460	50.8 ± 10.9 years	6 weeks	Intralesional pulsed radiofrequency treatment (RF)	Pain: Wong-Baker Scale. Function: AOFAS Ankle-Hindfoot Score	No serious complications. No significant adverse effects
Erden et al., 2020 [23]	217	CSI: 45 ± 9.1, ESWT: 43 ± 9.6, RTL: 41 ± 10.1	1, 3, 6 months	Group 1: Corticosteroid injection (CSI). Group 2: ESWT. Group 3: Radiofrequency thermal lesioning (RTL)	Pain: VAS. Evaluations at 1, 3, and 6 months	No complications in any treatment group
Huang et al., 2018 [16]	43	Median of 45 years	3, 6, 12 months	Group 1: Radiofrequency microtenotomy. Group 2: Gastrocnemius recession. Group 3: Combined treatment	Function: AOFAS, SF-36. Satisfaction and expectations. Vitality and recovery comparison	No serious postoperative complications. Greater vitality improvement in combined group (SF-36)
Lucas et al., 2015 [24]	61	46.9 ± 11.0 years (19–74 years)	33.3 months (16.1–46.6 months)	Bipolar percutaneous radiofrequency microtenotomy for plantar fascia release	Pain: VAS. Foot function: FFI. Patient satisfaction survey	No surgical complications. 1 revision surgery (1.6%). 1 DVT (1.6%). 2 pressure ulcers (3.3%)
Møller et al., 2022 [25]	70	≥18 years	1, 3, 6, 12 months	Group 1: Heavy slow resistance training (HSRT). Group 2: Radiofrequency microtenotomy (RFM)	Primary: Pain score (FHSQ-DK) at 6 months. Secondary: Other FHSQ-DK domains, GPE, IPAQ, PASS	Minimal complications with RFM. Monitoring for adverse effects, adherence, barriers
Ozan et al., 2016 [26]	56	ESWT: 46.03 years (25–62), RTL: 48.94 years (39–76)	1, 3, 6 months	Group 1: ESWT. Group 2: RTL	Pain: VAS. Function: Modified Roles-Maudsley Scale (RM)	No complications in any treatment group
Shah et al., 2016 [14]	3 patients (4 feet)	40–51 years	12 months	Ultrasound-guided radiofrequency coblation with lateral heel approach	Pain and function improvement. Real-time microdebridement visualization	No complications. Preserved foot biomechanics and proper healing
Wang et al., 2017 [27]	34	Median of 47 years	3, 6, 12 months	Group 1: Endoscopic plantar fasciotomy. Group 2: Open radiofrequency microtenotomy	Function: AOFAS, SF-36. Satisfaction and expectations	No serious complications. Persistent and recurrent pain in some patients
Wu et al., 2017 [28]	36	49.45 ± 9.90 years	1, 4, 8, 12 weeks	Group 1: Pulsed radiofrequency stimulation (PRF). Group 2: Lidocaine injection (control)	Pain: VAS for first step and general pain. Function: AOFAS. Plantar fascia thickness	No severe complications. Some temporary plantar numbness in control group
Yuan et al., 2020 [29]	31	52.13 years (24–77 years)	58.77 months	Group 1: Open plantar fascia release. Group 2: Percutaneous radiofrequency ablation	Pain: VAS. Function: AOFAS-AH. Recovery and surgical time	No severe complications. Two unsatisfied open release patients
Koh et al., 2022 [30]	128	Group A: 47.5 ± 11.1, Group B: 44.9 ± 12.9	6, 24 months	Group A: Radiofrequency coblation. Group B: Coblation with endoscopic gastrocnemius recession	Pain: VAS. Function: AOFAS hindfoot score, SF-36, Satisfaction and expectations	No wound complications. Persistent pain in some patients. 1 case of tarsal tunnel syndrome in Group B

**Table 2:** Results for the Joanna Briggs Institute (JBI) critical appraisal tool for case series studies.

Author/Year	it. 1	it. 2	it. 3	it. 4	it. 5	it. 6	it. 7	it. 8	it. 9	it. 10	Overall Quality
Colberg et al., 2022 [17]	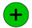	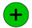	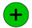	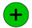	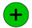	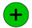	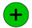	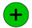	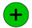	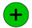	High
Colberg et al., 2019 [18]	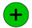	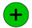	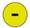	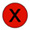	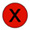	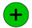	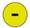	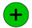	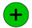	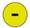	Moderate
Bagali et al., 2016 [20]	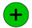	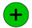	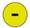	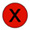	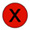	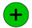	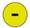	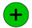	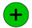	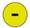	Moderate
Shah et al., 2016 [14]	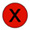	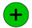	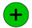	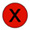	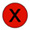	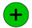	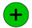	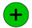	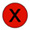	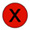	Low

Note: it. 1 = Were there clear criteria for inclusion in the case series? it. 2 = Was the condition measured in a standard, reliable way for all participants included in the case series? it. 3 = Were valid methods used for identification of the condition for all participants included in the case series? it. 4 = Did the case series have consecutive inclusion of participants? it. 5 = Did the case series have complete inclusion of participants? it. 6 = Was there clear reporting of the demographics of the participants in the study? it. 7 = Was there clear reporting of clinical information of the participants? it. 8 = Were the outcomes or follow-up results of cases clearly reported? it. 9 = Was there clear reporting of the presenting sites’/clinics’ demographic information? and it. 10 = Was statistical analysis appropriate? “Yes” was represented by the color green, “Unclear” by yellow, and “No” by red.

**Table 3:** Risk of bias in non-randomized studies–of interventions.

Autohr/Year	it. 1	it. 2	it. 3	it. 4	it. 5	it. 6	it. 7	it. 8
Koh et al., 2022 [30]	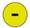	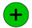	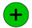	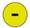	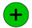	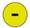	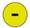	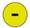
Yuan et al., 2020 [29]	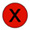	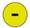	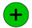	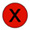	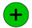	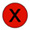	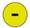	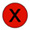
Eke et al., 2021 [22]	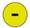	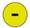	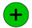	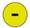	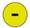	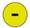	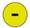	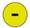
Erden et al., 2020 [23]	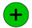	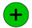	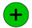	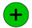	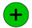	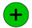	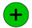	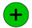
Chou et al., 2016 [21]	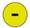	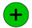	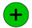	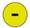	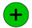	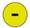	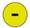	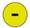
Lucas et al., 2015 [24]	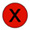	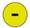	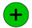	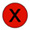	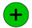	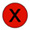	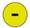	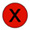
Ozan et al., 2016 [26]	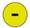	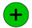	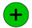	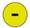	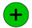	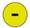	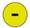	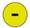
Wang et al., 2017 [27]	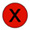	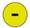	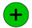	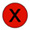	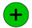	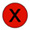	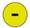	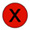
Huang et al., 2018 [16]	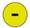	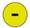	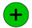	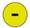	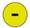	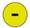	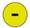	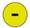

**Note:** it. 1 = Was there bias due to confounding variables that were not adequately controlled? it. 2 = Was there bias in the selection of participants, meaning that the groups compared were not appropriately matched or representative? it. 3 = Was there bias in the classification of interventions, ensuring that exposures or treatments were correctly assigned? it. 4 = Was there bias due to deviations from Intended Interventions, where participants did not adhere to the assigned treatment protocol? it. 5 = Was there bias due to Missing Data, meaning that data loss could influence the results? it. 6 = Was there bias in the measurement of outcomes, where the methods used for outcome assessment may have introduced systematic error? it. 7 = Was there bias in the selection of reported results, meaning that only certain outcomes were selectively reported? it. 8 = Was the overall risk of bias evaluated, considering all domains together?

**Table 4:** ROB-2 (Risk of bias in randomized trials).

Author/Year	it. 1	it. 2	it. 3	it. 4	it. 5	it. 6
Wu et al., 2017 [28]	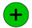	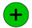	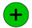	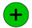	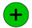	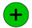
Møller et al., 2022 [25]	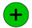	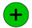	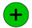	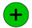	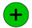	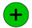

Note: it. 1 = Bias due to randomization—Was the randomization process conducted appropriately to ensure comparability between groups? it. 2 = Bias due to deviations from Intended Interventions—Were there deviations from the assigned intervention that could affect the study’s validity? it. 3 = Bias due to Missing Data—Was Missing Data handled appropriately, or could its absence introduce bias in the results? it. 4 = Bias in Measurement of Outcomes—Were outcome assessments conducted in a way that minimized measurement bias? it. 5 = Bias in Selection of Reported Results—Was there selective reporting of results, leading to potential misrepresentation of findings? it. 6 = Overall Risk of Bias—Final assessment considering the cumulative effect of all bias domains. Interpretation: “Low” risk of bias was represented by the color green, indicating minimal concerns regarding the study’s validity.”

**Table 5:** GRADE quality assessment.

Author(s) & Year	Study Design	Risk of Bias	Inconsistency	Indirectness	Imprecision	Publication Bias	Overall GRADE Assessment
Shah et al., 2016 [14]	Case Series	High	Unclear	Moderate	High	Unclear	Low
Colberg et al., 2022 [17]	Case Series	Moderate	Low	Low	Moderate	Low	Moderate
Colberg et al., 2019 [18]	Case Series	Moderate	Moderate	Low	Moderate	Moderate	Low
Koh et al., 2022 [30]	Cohort Study	Moderate	Low	Low	Moderate	Low	Moderate
Yuan et al., 2020 [29]	Comparative Study	Moderate	Moderate	Moderate	High	Moderate	Low
Eke et al., 2021 [22]	Retrospective Study	High	High	Moderate	High	Moderate	Low
Erden et al., 2020 [23]	Cohort Study	Moderate	Low	Low	Moderate	Low	Moderate
Wu et al., 2017 [28]	Randomized Controlled Trial	Low	Low	Low	Low	Low	High
Chou et al., 2016 [21]	Cohort Study	Moderate	Low	Low	Moderate	Low	Moderate
Lucas et al., 2015 [24]	Retrospective Study	High	High	Moderate	High	Moderate	Low
Ozan et al., 2016 [26]	Prospective Comparative Study	Moderate	Low	Low	Moderate	Low	Moderate
Bagali et al., 2016 [20]	Case Series	Moderate	Moderate	Low	Moderate	Moderate	Low
Møller et al., 2022 [25]	Randomized Controlled Trial	Low	Low	Low	Low	Low	High
Wang et al., 2017 [27]	Retrospective Comparative Study	Moderate	Moderate	Moderate	High	Moderate	Low
Huang et al., 2018 [16]	Retrospective Study	High	High	Moderate	High	Moderate	Low

## Data Availability

Data are contained within this article.

## Supplementary Materials

The following supporting information can be downloaded at: https://www.mdpi.com/article/10.3390/jcm14082843/s1, PRISMA 2020 Checklist [19].

## Abbreviations

The following abbreviations are used in this manuscript:

Abbreviation	Meaning
AOFAS	American Orthopaedic Foot & Ankle Society
BMI	Body Mass Index
CRD	Centre for Reviews and Dissemination
CSI	Corticosteroid Injection
DVT	Deep Vein Thrombosis
ESWT	Extracorporeal Shock Wave Therapy
FAAMA	Foot and Ankle Ability Measure—Activities of Daily Living
FAAMS	Foot and Ankle Ability Measure—Sports
FADI	Foot and Ankle Disability Index
FHSQ-DK	Foot Health Status Questionnaire—Danish Version
GPE	Global Perceived Effect
GRADE	Grading of Recommendations, Assessment, Development, and Evaluation
HSRT	Heavy Slow Resistance Training
IPAQ	International Physical Activity Questionnaire
JBI	Joanna Briggs Institute
NRS	Numeric Rating Scale
PASS	Patient Acceptable Symptom State
PRISMA	Preferred Reporting Items for Systematic Reviews and Meta-Analyses
PRP	Platelet-Rich Plasma
RF	Radiofrequency
RFM	Radiofrequency Microtenotomy
ROB-2	Risk Of Bias In Randomized Trials
ROBINS-I	Risk Of Bias In Non-randomized Studies—of Interventions
RTL	Radiofrequency Thermal Lesioning
SF-36	Short Form Health Survey
VAS	Visual Analog Scale

## Appendix A

### Appendix A.1. Search Strings for Electronic Databases

The original search string was created using PubMed. The search strings employed for Web of Science and MEDLINE via PubMed were translated using an automatic online tool https://www.sr-accelerator.com/#/polyglot: accessed on 10 February 2025.

#### Appendix A.1.1. Search Strategy for PubMed

URL: https://pubmed.ncbi.nlm.nih.gov (accessed on 5 February 2025).

(“fasciitis, plantar”[MeSH Terms] OR “Tibial Neuropathy”[MeSH Terms] OR (“plantar fasciitis”[Title/Abstract] OR “plantar fasciopathy”[Title/Abstract] OR “plantar fasciosis”[Title/Abstract])) AND (“Radiofrequency Therapy”[MeSH Terms] OR “Pulsed Radiofrequency Treatment”[MeSH Terms] OR (“radiofrecuency”[Title/Abstract] OR “microtenotomy”[Title/Abstract] OR “topaz”[Title/Abstract] OR “cobla-tion”[Title/Abstract])).

#### Appendix A.1.2. Search Strategy for Web of Science

URL: https://www.webofscience.com/wos/alldb/basic-search (accessed on 05 February 2025).

Filters: No filters were applied.

“plantar fasciitis” OR “heel pain” OR “plantar fasciosis” OR “plantar fasciopathy” (Topic) and “radiofrequency” “microtenotomy” OR “topaz” OR “coblation” OR “rf” (Topic).

#### Appendix A.1.3. Search Strategy for Scopus

(TITLE-ABS-KEY ((“plantar fasciitis” OR “plantar fasciosis” OR “plantar fasciopathy” OR “heel pain”)).

AND TITLE-ABS-KEY ((“radiofrequency” OR “microtenotomy” OR “Topaz” OR “coblation” OR “RF”))).

#### Appendix A.1.4. Search EBSCOH

(MH “Heel Pain”) OR (MH “Plantar Fasciitis”) OR TI ( plantar fasciitis or plantar heel pain or plantar fasciopathy or plantar fasciosis) OR AB ( plantar fasciitis or plantar heel pain or plantar fasciopathy or plantar fasciosis) AND (MH “Radiofrequency Thera-py+”) OR TI (radiofrequency therapy OR topaz OR microtenotomy OR coblation) OR AB (radiofrequency therapy OR topaz OR microtenotomy OR coblation).
